# Electrical Stimulation of the Mesencephalic Locomotor Region Attenuates Neuronal Loss and Cytokine Expression in the Perifocal Region of Photothrombotic Stroke in Rats

**DOI:** 10.3390/ijms20092341

**Published:** 2019-05-11

**Authors:** Michael K. Schuhmann, Guido Stoll, Arne Bohr, Jens Volkmann, Felix Fluri

**Affiliations:** Department of Neurology, University Hospital of Würzburg, 97080 Würzburg, Germany; Schuhmann_M@ukw.de (M.K.S.); Stoll_G@ukw.de (G.S.); Arne.Bohr@gmx.de (A.B.); Volkmann_J@ukw.de (J.V.)

**Keywords:** photothrombotic stroke, deep brain stimulation, mesencephalic locomotor region, neuroprotection, neuronal apoptosis, neuroinflammation

## Abstract

Deep brain stimulation of the mesencephalic locomotor region (MLR) improves the motor symptoms in Parkinson’s disease and experimental stroke by intervening in the motor cerebral network. Whether high-frequency stimulation (HFS) of the MLR is involved in non-motor processes, such as neuroprotection and inflammation in the area surrounding the photothrombotic lesion, has not been elucidated. This study evaluates whether MLR-HFS exerts an anti-apoptotic and anti-inflammatory effect on the border zone of cerebral photothrombotic stroke. Rats underwent photothrombotic stroke of the right sensorimotor cortex and the implantation of a microelectrode into the ipsilesional MLR. After intervention, either HFS or sham stimulation of the MLR was applied for 24 h. The infarct volumes were calculated from consecutive brain sections. Neuronal apoptosis was analyzed by TUNEL staining. Flow cytometry and immunohistochemistry determined the perilesional inflammatory response. Neuronal apoptosis was significantly reduced in the ischemic penumbra after MLR-HFS, whereas the infarct volumes did not differ between the groups. MLR-HFS significantly reduced the release of cytokines and chemokines within the ischemic penumbra. MLR-HFS is neuroprotective and it reduces pro-inflammatory mediators in the area that surrounds the photothrombotic stroke without changing the number of immune cells, which indicates that MLR-HFS enables the function of inflammatory cells to be altered on a molecular level.

## 1. Introduction

Considerable progress has been achieved in the treatment of acute ischemic stroke by the introduction of mechanical thrombectomy [1]. Nevertheless, ischemic stroke remains the most frequent cause of chronic disability in adults worldwide [2]. Around 86% of all stroke survivors present permanent limb paresis, and up to 11% are no longer able to walk, even after intensive physical training [3]. This might be due to limited neuroplasticity, but it might also be a result of local processes around the infarcted brain area, such as neuroinflammation. Recently, we have shown that high-frequency stimulation (HFS) of the mesencephalic locomotor region (MLR) enables gait restoration after photothrombotic lesioning of the sensorimotor cortex in rats [4]. In line with these findings, pharmacological or electrical stimulation of the MLR triggers locomotor behavior in decerebrated animals, such as cats [5], rats [6], and nonhuman primates [7]. Additionally, MLR stimulation has been applied in patients with Parkinson disease [8,9,10]. Anatomically, the MLR overlaps the pedunculopontine tegmental nucleus, cuneiform nucleus, and mescencephalic reticular nucleus [11], and it is indirectly connected to the cerebral cortex. HFS may elicit glutamatergic neurons within the MLR, which promotes locomotion, but might also stimulate other cell types within the electrical field of the electrode. Thus, the stimulation of the MLR might modulate neural circuits that are involved in non-motor functions, as has been shown for cardiovascular and sympathetic/autonomic regulation in rodents [12]. Whether invasive MLR-HFS also intervenes in neuro-immune communication has not yet been examined. Interestingly, there are only few studies that investigate whether non-invasive brain stimulation modulates cerebral inflammation after brain injury [13,14]. Inflammatory processes play a crucial role in the pathogenesis of stroke, and thus provide a promising target for current translational research [15]. Early post-ischemic inflammation is characterized by the activation of platelets, endothelial cells [16], and local microglia [17]. The upregulation of pro-inflammatory cytokines and infiltration of neutrophils, macrophages, and lymphocytes exacerbate further ischemic tissue damage [18]. Additionally, T cells can be detected in the ischemic cerebral tissue as early as 3 h after infarction [18,19,20,21]. Human trials for immunomodulatory drug therapies in the (sub)acute phase of ischemic stroke have yielded limited translational success in Caucasian patients [22,23], because of their systemic activity, among other reasons. In contrast, deep brain stimulation (DBS) is an anatomically-confined therapy; overall, it has anti-inflammatory effects when applied in the fastigial nucleus [24] or intracortically in the perilesional area after ischemic stroke [25]. We examined the effect of MLR-HFS on apoptotic and inflammatory processes in the peri-infarcted brain region (sensorimotor cortex) of male Wistar rats within the first 24 h after stroke onset, with these considerations in mind.

## 2. Results

### 2.1. No Change of Infarct Size due to MLR-HFS

**Figure 1:**
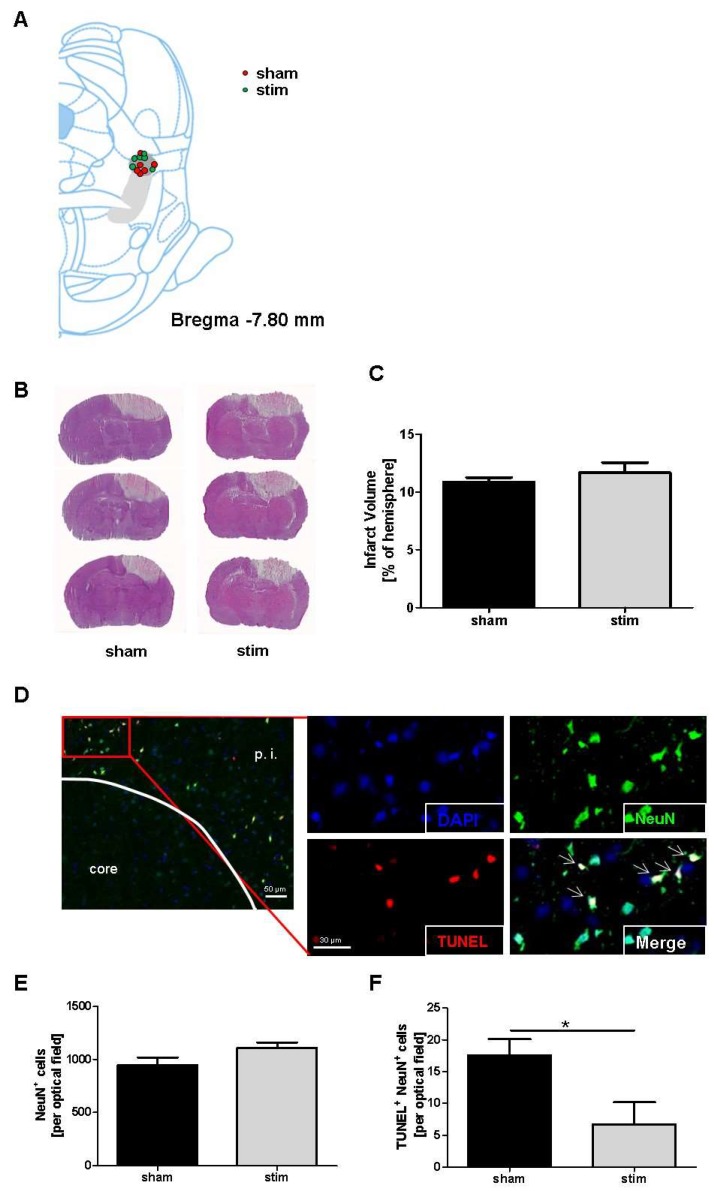
Stimulation of the mesencephalic locomotor region in the acute phase after photothrombotic stroke does not alter lesion size but exhibits neuroprotection. (**A**), Schematic reconstructions of electrode placements. The light gray area indicates the pedunculopontine tegmental nucleus, the dark gray area the cuneiform nucleus. (**B**), Representative hematoxylin and eosin stains of three corresponding brain sections of an unstimulated rat (sham) and a rat stimulated with 40 μA (stim) 27 h after a stroke. (**C**), Infarct volumes are similar (*n* = 6/group) between the two treatment groups. Unpaired, two-tailed Student’s *t*-test. (**D**), Representative brain section stained for the neuronal marker NeuN (green) and subjected to TUNEL assay (red) to visualize apoptosis. Nuclei of the cells are counterstained with DAPI (blue). p. i. indicates peri-infarct area. Arrows indicate NeuN, TUNEL double positive cells. 30 µm scale bar counts for the four high magnification images. (**E**), Quantification of neuronal density per optical field in the cortical photothrombotic penumbra at day 1 (*n* = 6/group). (**F**), Quantification of apoptotic neurons (NeuN, TUNEL double positive cells) per optical field in the cortical photothrombotic penumbra at day 1 (*n* = 6/group). Unpaired, two-tailed Student’s *t*-test. * *p* < 0.05.

In a first set of experiments, we assessed whether MLR-HFS in rats had an impact on lesion size after photothrombotic stroke. HE staining yielded a slight variability of electrode placement in relation to midbrain structures, with non-visible tissue damage, except for the electrode tracks toward the MLR. As shown in Figure 1A, most of the electrode tips were situated close to the cuneiform nucleus. Only one animal revealed a placement of electrode outside the MLR, and it was thus excluded.

The infarct volumes were assessed by planimetry of HE-stained brain sections in sham-stimulated rats and in rats undergoing MLR-HFS for 24 h, beginning 3 h after induction of photothrombotic stroke. Both the sham and the stimulated group showed a similar size of infarct volume within the sensorimotor cortex (sham vs stim: 10.85 ± 0.41% vs 11.67 ± 0.89%; *p* > 0.05) (Figure 1B,C).

### 2.2. Reduction of Perilesional Neuronal Apoptosis after MLR-HFS

The neuronal marker neuron-specific nuclear protein (NeuN) was immunostained in samples of cerebral tissue to assess whether MLR-HFS applied for 24 h protects neurons from apoptosis after photothrombotic stroke. In detail, five consecutive coronal brain tissue slices that were localized at the stereotaxic level of the bregma (i.e., where the caudate putamen is also localized) were analyzed; in each of these slices, five regions of interest around the lesion were selected. When comparing neuronal density in both the stimulated and sham-stimulated animals, no significant difference was found regarding neuronal density in the perilesional area (sham vs stim: 946.8 ± 72.5 vs 1111.0 ± 48.5 cells/optical field; *p* > 0.05) (Figure 1D,E). In a next step, neuronal survival was examined while using a maker for apoptosis (TUNEL). This analysis yielded a significant reduction of apoptotic neurons in rats undergoing MLR-HFS for 24 h when compared to the control group (sham vs stim: 17.5 ± 2.6 vs 6.7 ± 3.5 cells/optical field; *p* < 0.05) (Figure 1F).

### 2.3. Attenuation of Perilesional Interleukine/Chemokine Concentration after MLR-HFS

**Figure 2:**
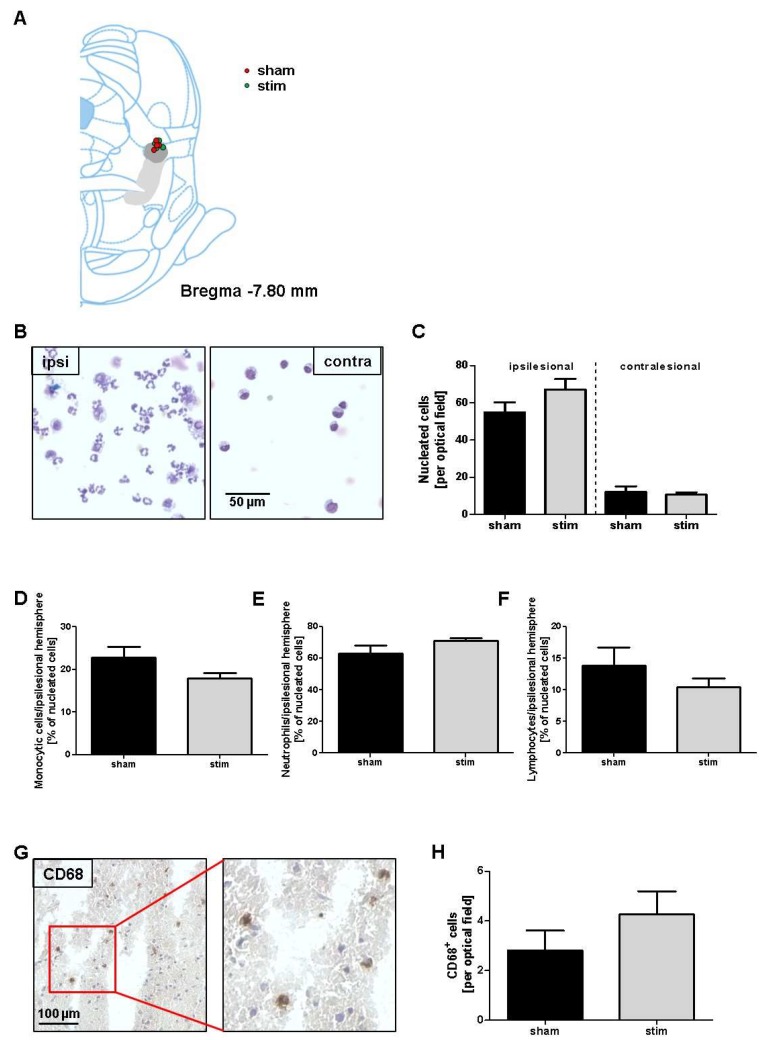
Stimulation of the mesencephalic locomotor region does not modulate photothrombosis-driven immune cell recruitment to the brain. (**A**), Schematic reconstructions of electrode placements. The light gray area indicates the pedunculopontine tegmental nucleus, the dark gray area the cuneiform nucleus. (**B**), Representative Pappenheim staining within brain cytospin preparations of the ipsilesional (ipsi) and contralesional (contra) hemispheres of unstimulated (sham) and high-frequency stimulated rats (stim). (**C**–**F**), Quantification revealed comparable numbers of nucleated cells (**B**), monocytic cells (**C**), neutrophils (**D**), and lymphocytes (**E**) 27 h after photothrombosis within the two treatment groups (*n* = 4/group). (**G**), Representative immunocytologic staining of CD68+ cells within an ischemic rat brain section (**left**) together with a high magnification of the representative staining as indicated by the red square (**right**). (**H**), Quantification revealed comparable numbers of CD68+ monocytes in the ipsilesional hemispheres of unstimulated (sham) and high-frequency stimulated (stim) rats 27 h after photothrombosis (*n* = 5–6/group). Unpaired, two-tailed Student’s *t*-test.

During the development of brain infarction, external signals from the cerebral microenvironment, in concert with intrinsic signaling pathways, determine whether neurons will die. In this context, inflammatory processes play a crucial role, namely the invasion of immune cells due to ischemia and beginning necrosis of cerebral tissue. Depending on the cell type and its phenotype, the immune cells may have preferentially more anti-inflammatory or pro-inflammatory effects [26]. Therefore, we investigated whether MLR-HFS alters the composition of the cellular infiltrates around the photothrombotic lesion. For this purpose, we generated the cytospins of brain tissue dissected 0–4 mm anterior from the bregma. In both stimulated and unstimulated rats, leukocytes were significantly more abundant in the lesioned brain hemisphere (sham vs stim: 54.9 ± 5.4 vs 67.2 ± 5.6 cells; *p* > 0.05) when compared to the contralateral side (sham vs stim: 12.2 ± 2.9 vs 10.8 ± 1.2 cells; *p* > 0.05) (Figure 2B,C). Further examinations were then focused on subpopulations of brain-invading immune cells in the border zone of the photothrombotic lesion, namely monocytes, neutrophils, and lymphocytes. However, the amounts of monocytes (sham vs stim: 22.9 ± 2.4% vs 18.0 ± 2.4%; *p* > 0.05), neutrophils (sham vs stim: 67.9 ± 5.1% vs 71.1 ± 1.6%; *p* > 0.05), and lymphocytes (sham vs stim: 13.8 ± 2.9% vs 11.0 ± 1.4%; *p* > 0.05) were similar in the stimulated and control groups (Figure 2D–F). The same was also true for CD68+ immune cells (i.e., microglia and macrophages) in the peri-infarcted area of stimulated and unstimulated animals (sham vs stim: 2.8 ± 0.8 vs 4.3 ± 0.9 cells/optical field; *p* > 0.05) (Figure 2G,H).

**Figure 3:**
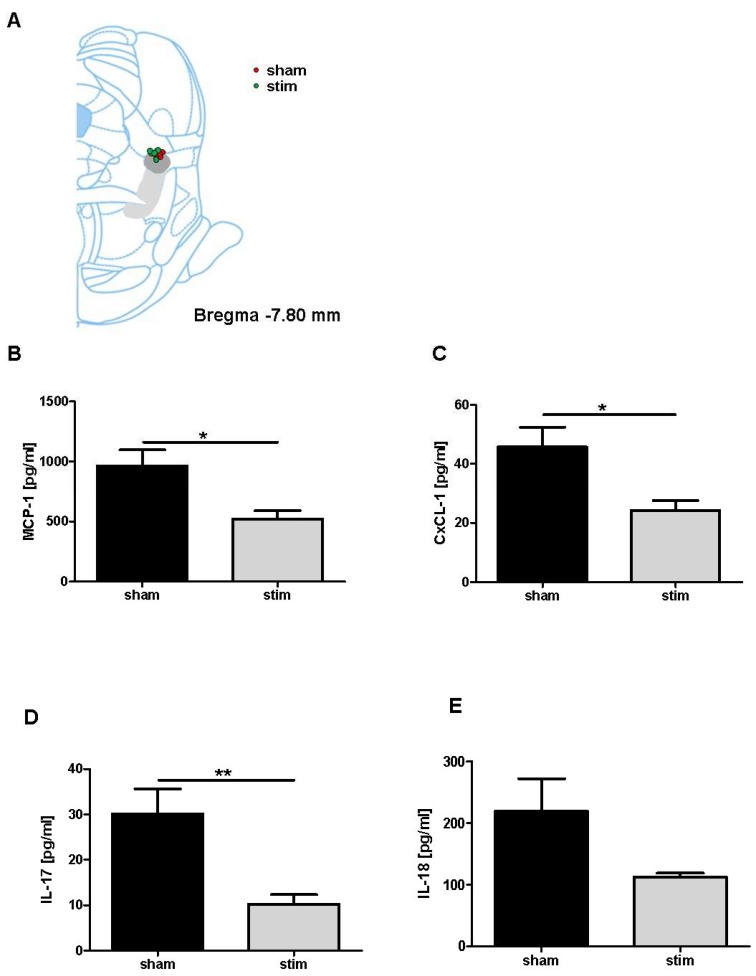
Stimulation of the mesencephalic locomotor region attenuates the perilesional concentration of interleukins and chemokines. Schematic reconstructions of electrode placements. The light gray area indicates the pedunculopontine nucleus, the dark gray area the cuneiform nucleus (**A**). Absolute amounts of monocyte chemotactic protein-1 (MCP-1) (**B**), chemokine (C-X-C motive) ligand 1 (CxCL-1) (**C**), interleukin (IL)17 (**D**), and IL-18 (**E**) were quantified in slices of the perilesional sensorimotor cortex 1 mm before the bregma in unstimulated (sham) and high-frequency stimulated rats (stim) 27 h after photothrombotic stroke (*n* = 5–6/group). Unpaired, two-tailed Student’s t-test. * *p* < 0.05; ** *p* < 0.01.

Next, we investigated whether MLR-HFS is able to modulate the activation of immune cells in terms of their capacity to synthesize cytokines and chemokines. Triggered by oxidative stress and pro-inflammatory agents, monocytes/macrophages produce MCP-1 [27] and secrete IL-18 [28]. When compared to sham-stimulated rats, animals undergoing MLR-HFS for 24 h exhibited a significant decrease in MCP-1 and reduced levels of IL-18 in the border zone of the photothrombotic lesion (MCP-1: 962.9 ± 133.0 (sham) vs 520.1 ± 69.69 pg/mL (stim); *p* < 0.05. IL-18: 219.3 ± 52.7 (sham) vs 112.7 ± 6.1 pg/mL (stim); *p* = 0.05). In addition, the neutrophils/macrophages synthesize CXCL-1 [29] and lymphocytes (namely γδ T cells) release IL-17, which promote further tissue damage [30]. Again, measurements of local concentrations revealed significant reductions of CXCL-1 after MLR-HFS (CXCL-1: 45.7 ± 6.6 (sham) vs 24.2 ± 3.4 pg/mL (stim); *p* < 0.05. IL-17: 30.2 ± 5.5 (sham) vs 10.2 ± 2.1 pg/mL (stim); *p* < 0.05). (Figure 3A–D).

## 3. Discussion

This study yielded the following main findings: MLR-HFS applied in the early phase after photothrombosis (i) exerts a neuroprotective effect in the perilesional area around the photothrombotic infarction; (ii) does not alter the composition of immune cells; and, (iii) reduces the synthesis of cytokines and chemokines in the perilesional area of the photochemically-induced lesion.

A growing body of studies report that DBS, particularly of the subthalamic nucleus, has a neuroprotective effect on dopamine neurons of the substantia nigra in preclinical models of Parkinson’s disease [31,32]. There are also several studies demonstrating that DBS has a therapeutic effect in a rat stroke model [33,34]. DBS of other cerebral structures, such as the anterior thalamic nuclei [35] or fastigial nucleus [36], also resulted in neuroprotection. Similarly, as presented here, HFS of the MLR reduced neuronal apoptosis in the perilesional region after experimental stroke, which indicates, that even DBS of these remote cerebral regions results in neuroprotection. The exact mechanisms of DBS that lead to reduced apoptosis, and thus a neuroprotective effect, are not well understood. Although the MLR has no direct axonal projections to the sensorimotor cortex, it is nevertheless indirectly connected to the cortex via a relay in the thalamus [37]. There is some evidence that the brain-derived neurotrophic factor/tropomyosin receptor kinase type B signaling pathway is involved in the neuroprotective effect of electrical stimulation [32]. Another study suggests that electrical stimulation minimizes glutamate-mediated hyperexcitability, and thus neuronal cell death [38]. Recently, DBS of the fastigial nucleus has been shown to modulate apoptosis via an intracellular receptor, the peroxisome proliferator-activated receptor gamma (PPAR-γ). This ligand-modulated transcriptional factor has an anti-apoptotic function following ischemic stroke [39]. PPAR-γ was up-regulated and thereby prevented neuronal apoptosis when electrical stimulation was applied to the fastigial nucleus, [40].

Neuronal cell death also results from local inflammatory processes. The first immune cells that respond to cerebral tissue damage within minutes or hours after ischemic stroke are brain-intrinsic microglia, followed by leukocytes [18]. The leukocytes enter the ischemic brain area from cerebral blood vessels via the compromised blood–brain barrier [41]. The invasion of these immune cells into the infarcted area is guided by chemokines/cytokines that are derived from dying neurons [41]. The kinetics of leukocyte infiltration into the infarcted brain region of animal stroke models suggest that neutrophils migrate early in the course of ischemic stroke, followed by macrophages and natural killer cells, whereas the T and B lymphocytes invade the infarcted tissue later on [42]. Therefore, we investigated whether MLR-HFS alters leukocyte infiltration when started 3 h after induction of infarction and applied continuously for 24 h. No difference was found between stimulated and unstimulated animal regarding the cellular composition of immune cells after finishing MLR-HFS. This might be explained, in part, by the fact that most of the leukocyte subpopulations are present in the infarcted area as early as three hours after the induction of photothrombotic infarction. Additionally, the observation might also indirectly demonstrate that MLR-HFS does not exert a stabilizing effect on the blood-brain barrier, which allows for immune cells to continuously penetrate into the infarcted area.

Next, we analyzed whether MLR-HFS might alter immune cells in their capacity to synthesize pro-inflammatory agents. Being triggered by oxidative stress, monocytes and macrophages produce MCP-1, which regulates the migration and infiltration of monocytes, memory T lymphocytes, and natural killer cells [27]. In response to inflammatory mediators (e.g., tumor necrosis factor-α, interferon-γ), neutrophils and macrophages produce CXCL-1, which exerts neutrophil-chemoattractant activity during the early phase of ischemic stroke [29]. At this early stage, the cellular infiltrate also includes CD4^+^ and CD8^+^ T cells [43], as well as γδ T cells [30]; the latter promote further tissue damage by secreting IL-17 [30]. When we measured MCP-1, CXCL-1, and IL-17 in the perilesional region in both, stimulated and non-stimulated animals, the analyses revealed a significant reduction of these pro-inflammatory agents. This finding indicates that the electrical stimulation of the MLR reduces the synthesis and/or secretion of interleukins and chemokines of immune cells in areas far from the stimulation site. The mechanism(s) underlying these processes remains elusive. The principles of DBS are still poorly understood, but there is some evidence that HFS might silence neurons through overstimulation or depolarization, which in turn might be mediated by the inactivation of sodium channels [44,45]. Interestingly, immune cells also express voltage-gated sodium channels, namely macrophages [46], lymphocytes [47,48], and microglia [49]. A recently-published study suggests that the inflammatory response, especially of monocyte/macrophages, might be modulated via these voltage-gated sodium channels, and thus might also be involved in macrophage polarization and phenotype switch [50]. This effect is probably associated with the blockage of sodium channels, as has been demonstrated in an animal model of multiple sclerosis (experimental autoimmune encephalitis): when the sodium channels of macrophage/microglia were blocked after administration of phenytoin or when using mice lacking such cells, the activation of macrophages/microglia was significantly reduced [51]. DBS also probably induces such an inhibition of sodium channels [44]. This might be a possible explanation of how DBS intervenes in neuro-immune communication.

Whether HFS exerts a more neuroprotective and anti-inflammatory effect than stimulation in a low frequency range is another unanswered question. Several studies have addressed this issue. HFS of the hippocampus, subthalamic, and anterior thalamic nucleus diminished the apoptosis-related proteins and consequently reduced neuronal loss [32,35,52]. HFS of the anterior thalamic nucleus was further associated with the attenuated expression of pro-inflammatory cytokines [53]. These observations are in line with our findings. Low frequency intracortical stimulation close to the photochemically-induced infarction [38] and low frequency stimulation of the fastigial nucleus [54] also significantly reduced neuronal apoptosis in the perilesional area of infarction. However, beside the different stimulation sites and different disease models, the stimulating intensity ranged from 30 to 100 µA, while the duration of stimulation varied widely between these studies, which makes an accurate comparison difficult. Nevertheless, the literature suggests that there seems to be a lowest common denominator, which consists of a neuroprotective and even anti-inflammatory efficacy of DBS. Thus, further efforts are needed to characterize the underlying mechanisms of DBS that result in neuroprotection and anti-inflammatory effects.

In a clinical setting, one might argue whether using invasive approaches, such as DBS, would be conceivable during the acute phase of stroke. Here, we simply wanted to test whether DBS of the MLR during the acute phase of stroke might also exert non-motor effects, such as the modulation of neuroinflammatory processes. Thus, the present study is primarily a proof of principle. Therefore, whether this approach to neuromodulation will ever be introduced in acute stroke therapy is outside the scope of this study. In contrast to non-invasive stimulating methods, such as transcranial direct-current stimulation (tDCS) or transcranial magnetic stimulation (TMS), invasive stimulation using a microelectrode allows for more precise modulation of distinct brain targets that are involved in specific cerebral networks. Our experiments clearly show that MLR-HFS reduces the inflammatory processes in the acute phase after stroke, which may prevent the neuronal cells from further harm. As a consequence, the use of a less invasive neuromodulation procedure should be examined in the stroke models, since these methods might be of potential relevance during the acute phase of stroke. So far, there are only a few reports on the anti-inflammatory effects of non-invasive stimulating procedures after cerebral damage. Sasso and coworkers showed that, even remote stimulation with repetitive TMS exerts an anti-inflammatory effect and improves functional recovery in a model of focal brain injury, namely hemicerebellectomy [14]. Similar results were achieved in a rat stroke model when tDCS was applied in the acute phase of ischemia [13].

This study has several limitations. First, all of the experiments were conducted using HFS, whereas low frequency stimulation or different amplitudes were not tested. Even if these parameters may not elicit motor effects, they might have an effect on non-motor processes. However, the primary target of this study was to investigate whether electrical stimulation in the MLR triggers non-motor effects (i.e., proof of concept) and, if so, whether this is also true for stimulating parameters that enable motor processes. Further studies are now required to investigate the non-motor effects under different stimulating conditions. Second, we performed histological analyses only 24 h after starting MLR-HFS and did not test its effect within this time frame, which should be addressed in further studies. Third, charge density in the cerebral tissue was not determined. Of note, Ranck has characterized the current-distance relation in cerebral mammalian tissue [55]. According to his measurements, a stimulating amplitude of e.g., 50 µA impacts neuronal cell bodies within a radius of 250 µm and fibers within a radius of 700 µm.

## 4. Materials and Methods

### 4.1. Animals

Thirty-four male Wistar rats (Charles River, Sulzfeld, Germany), 10–12 weeks old, were used in this study. The rats were assigned to a group undergoing sham stimulation (*n* = 17) or MLR-HFS (*n* = 17) after the induction of photothrombosis. Two rats of the sham group were excluded, since no infarction was detected after harvesting the brain. In the group receiving MLR-HFS, one animal was excluded, since the microelectrode was not within the MLR.

Rats were acclimatized before intervention for one week in a room of our animal facility, with controlled temperature (22 ± 0.5 °C) under a 12 h/12 h light/dark cycle. They were allowed free access to water and food. The institutional review board of the Julius-Maximilians-University, Würzburg, and the local authorities at the Regierung von Unterfranken, Würzburg, Germany approved all of the animal experiments (TVA55.2-2531.01-102/13; 02.10.2013). The study was performed according to the ARRIVE guidelines (Animal Research: Reporting of In Vivo Experiments; https://www.nc3rs.org.uk/arrive-guidelines).

### 4.2. Induction of Photothrombotic Stroke

Photothrombotic stroke was induced in all rats, as described previously [4]. Briefly, the rats were fixed in a stereotactic frame under deep anesthesia (isoflurane 2.5%). A template with an aperture encompassing the right sensorimotor cortex (5 mm anterior to 5 mm posterior and 0.5 mm to 5.5 mm lateral to the bregma) was put on the exposed skull. A cold light source (Olympus KL1500LCD, Mainz, Germany) was positioned over the aperture. Rose Bengal (Sigma-Aldrich, Darmstadt, Germany) in NaCl 0.9% was intravenously injected and thereafter the determined cerebral region was illuminated for 15 min. During the whole procedure, body temperature was maintained at 37 ± 0.5 °C by a feedback-controlled heating system.

### 4.3. Microelectrode Implantation

Immediately after the induction of photothrombosis, a monopolar-stimulating microelectrode was implanted in the ipsilateral MLR (7.8 mm posterior, 2.0 mm lateral, and 5.8 mm ventral to the bregma), as recently reported in detail [4]. Briefly, a microelectrode (FHC Inc., Bowddoin, ME, USA) was inserted in the MLR and then attached to the skull using anchor screws and dental cement. A plug (GT-Labortechnik, Arnstein, Germany) was connected with the electrode and fixed with additional dental cement. The animals were allowed to wake up after suturing the wound edges.

### 4.4. High-Frequency Stimulation of the Mesencephalic Locomotor Region

HFS (frequency, 130 Hz; pulse length, 60 µs; monophasic square wave pulses) was applied to the MLR for 24 h using a stimulus generator three hours after inflicting a photochemical lesion in the sensorimotor cortex (STG 4002, Multichannel Systems, Reutlingen, Germany). The threshold current amplitude was assessed for each rat by assessing the locomotor behavior under MLR-HFS, as described recently [4]. The stimulating amplitude was determined, as follows: beginning with 20 µA, the intensity was increased in steps of 10 µA until maximal locomotion was observed. The lowest current evoking locomotion was chosen for the 24-h MLR-HFS. The animals were randomly allocated to a group undergoing MLR-HFS or sham stimulation (which served as control). No behavioral differences were found between the stimulated and sham-stimulated groups during the 24 h of the experiment.

MLR-HFS was started 3 h after photochemically-induced stroke for the following reason: inflammatory processes occur very early after ischemic stroke; within 12 h after cerebral ischemia, immune cells, such as neutrophils, are detected in the infarcted area [18], while the T cells have been detected as early as 24 h after stroke [20,21]. Based on these observations, we believe that an intervention protecting from inflammatory processes should start before these detrimental processes become effective. MLR-HFS was only performed for 24 h, since we hypothesized that stimulus-dependent suppression of inflammatory mechanisms for this period might be long enough to prevent the inflammatory processes from emerging, or at least to attenuate them.

### 4.5. Collection of Cerebral Tissue

Immediately after 24-h MLR-HFS (i.e., 27 h after induction of photothrombosis), the rats were anesthetized by administering pentobarbital and perfused transcardially with phosphate buffered saline for 4 min. Thereafter, the brains were removed. One millimeter anterior to the bregma, a 2-mm thick brain slice was cut. Cortex encompassing the perilesional area was dissected from the subcortical tissue for further analysis.

### 4.6. Brain Cell Separation

Brain tissue was mechanically dissociated, and the mononuclear cells were isolated from the interface of a 30–50% Percoll (Amersham Biosciences, Buckinghamshire, UK) density gradient, as described [56], followed by cytospin slide preparation.

### 4.7. Immunohistochemistry

Immunohistochemistry and histology of cryo-embedded brain slices (10-µm thick) were performed, as described elsewhere [57], using the antibodies anti-mouse CD68 (ab31630, Abcam) and anti-mouse NeuN (MAB377, Millipore, Darmstadt, Germany). For quantification of early apoptotic cell death, the In Situ Cell Death Detection Kit (12156792910, Roche, Mannheim, Germany) was used according to the user’s manual. Cell counting was performed using five subsequent slices (distance 100 µm) per animal. The number of CD68-positive cells and apoptotic neurons (TUNEL/NeuN double positive cells) was analyzed by counting five optical fields per cortex region. The sections were analyzed under a microscope (Nikon Eclipse 50i, Nikon, Amsterdam, Netherlands) that was equipped with a charge-coupled device camera, using 20-fold magnification.

For the quantification of infarct volumes, hematoxylin and eosin (HE) staining was performed (10-μm slices) using every tenth slide. 20 slices per animal were necessary to assess the infarct volume in its anterior-posterior extension. Quantification of brain infiltrating leukocytes was performed with Pappenheim-stained Cytospin preparations.

Brain slices (10-μm thick) encompassing the MLR were stained with HE to visualize the anatomic locations of the electrode tip.

### 4.8. Cytokine/Chemokine Quantification

We quantified the production of interleukin (IL)-17, IL-18, monocyte chemotactic protein-1 (MCP-1), and chemokine (C-X-C motive) ligand 1 (CxCL-1) in rat brain lysates while using a fluorescent bead immunoassay (LEGENDplex, Biolegend, San Diego, CA, USA), according to the manufacturer’s instructions.

### 4.9. Statistical Analysis

For statistical analysis, the GraphPad Prism 5.0 software package (GraphPad, San Diego, CA, USA) was used. The results are given as mean ± standard error of the mean. Data were tested for Gaussian distribution with the D’Agostino and Pearson omnibus normality test, and then analyzed using the unpaired, two-tailed Student’s t-test. *p* < 0.05 was considered to be statistically significant.

## 5. Conclusions

MLR-HFS reduces neuronal apoptosis, i.e., the loss of neurons in the perilesional area of a photochemically-induced infarct within the sensorimotor cortex. Additionally, MLR-HFS attenuates pro-inflammatory cytokines and chemokines, while the number of immune cells remains unchanged. This suggests that MLR-HFS might influence the functionality and probably alter the (pro-inflammatory) phenotype (M1) of these cells when applied in the subacute course of stroke (i.e., beginning within 3 h after the induction of photothrombosis). Notably, most of the immune cells exhibit pro-inflammatory properties in the first hours after stroke onset. Thereafter, some subpopulations of these immune cells are able to change to an anti-inflammatory and neuroprotective phenotype (M2), which probably indicate that electrical stimulation might have its most beneficial effect early and within a narrow window after stroke onset.

## Abbreviations

CxCL-1	chemokine (C-X-C motive) ligand 1
DBS	deep brain stimulation
HFS	high-frequency stimulation
IL	interleukin
MCP-1	monocyte chemotactic protein-1
MLR	mesencephalic locomotor region
PPAR-γ	peroxisome proliferator-activated receptor gamma
tDCS	transcranial direct-current stimulation
TMS	transcranial magnetic stimulation
